# Report from the World Health Organization’s immunization and vaccines-related implementation research advisory committee (IVIR-AC) meeting, virtual gathering, 26 February–1 March 2024

**DOI:** 10.1016/j.vaccine.2024.04.057

**Published:** 2024-05-31

**Authors:** Philipp Lambach, Sheetal Silal, Alyssa N. Sbarra, Mitsuki Koh, Rakesh Aggarwal, Habib Hasan Farooqui, Stefan Flasche, Alexandra B. Hogan, Sun-Young Kim, Kathy Leung, William J. Moss, Patrick K. Munywoki, Allison Portnoy, Meru Sheel, Xuan-Yi Wang

**Affiliations:** a Immunizations, Vaccines and Biologicals, World Health Organization, Geneva, Switzerland; b Modelling and Simulation Hub, Africa, University of Cape Town, Cape Town, South Africa; c Centre for Global Health, Nuffield Department of Medicine, Oxford University, Oxford, United Kingdom; d Johns Hopkins Bloomberg School of Public Health, Baltimore, MD, United States; e Jawaharlal Institute of Postgraduate Medical Education & Research, Puducherry, India; f College of Medicine, QU Health, Qatar University, Qatar; g Centre for Global Health, Charite, Berlin, Germany; h School of Population Health, University of New South Wales, Sydney, Australia; i Seoul National University, Seoul, South Korea; j School of Public Health, The University of Hong Kong, Hong Kong Special Administrative Region; k Kenya Medical Research Institute, Centre for Global Health Research, Nairobi, Kenya; l Department of Global Health, Boston University School of Public Health, Boston, United States; m Center for Health Decision Science, Harvard T.H. Chan School of Public Health, Boston, United States; n School of Public Health, Faculty of Medicine and Health, University of Sydney, Sydney, Australia; o Shanghai Institute of Infectious Disease and Biosecurity, Fudan University, Shanghai, China

**Keywords:** IVIR-AC, Malaria, Economic evaluation, Meta-analysis, EPI

## Abstract

The Immunization and Vaccine-related Implementation Research Advisory Committee (IVIR-AC) is the World Health Organization’s key standing advisory body to conduct an independent review of research, particularly of transmission and economic modeling analyses that estimate the impact and value of vaccines. From 26th February–1st March 2024, at its first of two semi-annual meetings, IVIR-AC provided feedback and recommendations across four sessions; this report summarizes the proceedings and recommendations from that meeting. Session topics included modeling of the impact and cost-effectiveness of the R21/Matrix-M malaria vaccine, *meta*-analysis of economic evaluations of vaccines, a global analysis estimating the impact of vaccination over the last 50 years, and modeling the impact of different RTS,S malaria vaccine dose schedules in seasonal settings.

## 1 Main text

### 1.1 Context

Robust, evidence-based policy should inform vaccination programs. Serving as an advisory body to the World Health Organization (WHO), the Immunization and Vaccines-related Implementation Research Advisory Committee (IVIR-AC) evaluates and reviews various vaccine effectiveness and impact studies, value assessments, and modeling analyses related to priority topics for the Strategic Advisory Group of Experts on Immunization (SAGE) and the Immunization, Vaccines, and Biologicals (IVB) Department. The main role of IVIR-AC is to provide advice and recommendations to WHO through topic-based sessions presented during semi-annual meetings. The committee has no executive or decision-making power [1]. This report summarizes proceedings and recommendations of the IVIR-AC’s virtual meeting, 26th February–1st March 2024[2].

### 1.2 Scope and objectives of meeting

WHO IVB and SAGE requested four meeting sessions to cover various topics including: modeling of the impact and cost-effectiveness of the R21/Matrix-M malaria vaccine; meta-analysis of economic evaluations of vaccines; global analysis estimating the impact of vaccination over the last 50 years; and modeling the impact of different RTS,S malaria vaccine dose schedules in seasonal settings. Session objectives, committee feedback, and recommendations are described for each session below.

### 1.3 Summary of sessions

#### 1.3.1 Session 1: Modeling public health impact and cost-effectiveness of the R21/Matrix-M malaria vaccine

Since October 2023, WHO has recommended two vaccines (RTS,S/AS01 [3] and R21/Matrix-M [4]) to prevent *Plasmodium falciparum* malaria in children. Phase III trials of R21/Matrix-M vaccine have shown high vaccine efficacy and no major safety concerns [5]. Also, R21/Matrix-M is expected to be manufactured at a lower cost and higher scale than RTS,S/AS01. However, individual countries, with varying transmission intensities, seasonal patterns, and existing packages of non-vaccine malaria interventions, will need to decide which vaccine to introduce among communities with high malaria risk. At the meeting, two research teams presented completed analyses and modeling plans using Phase II and III trial data to assess R21/Matrix-M vaccine impact and cost-effectiveness to help guide decision-making on the choice of optimal product for specific settings.

A modeling team from Imperial College London [6] presented results from analyses of Phase II and III trial data to estimate the impact and cost-effectiveness of R21/Matrix-M vaccine across different delivery strategies (i.e., age-based vaccination, seasonal vaccination, and a hybrid of these approaches), a range of transmission intensities and seasonal transmission settings (i.e., perennial and seasonal). The team fit a semi-mechanistic model to Phase IIb trial data to estimate the relationship between anti-circumsporozoite protein antibody titers and vaccine efficacy against clinical malaria. Then, the team used these model fits in a mathematical transmission model to estimate cases, deaths, and disability-adjusted-life-years (DALYs) averted and the cost-effectiveness of vaccination over a 15-year time horizon. IVIR-AC noted that the presented modeling represents an adequate framework to predict the impact of R21/Matrix-M based on the limited data available. Results suggested that the cost per DALY averted of R21/Matrix-M introduction is comparable to other existing malaria interventions and childhood vaccines. Additionally, cost-effectiveness was estimated to be higher at higher transmission intensities among all schedules; across seasonal settings, cost-effectiveness estimates were also similar across dosing schedules. Modeled estimates across lower transmission intensity settings were outside confidence bounds for non-seasonal Phase III trial sites, suggesting unexplained uncertainty in these settings (i.e., with lower transmission intensity). IVIR-AC recommends that the work supports the use of R21/Matrix-M in settings including those with low transmission; however, predictions are to be treated with some caution as the predictions extend well beyond the observed data and because of the potential mismatch of its results with the observations in the clinical trial of low point estimates of vaccine efficacy in low transmission and no-seasonal-malaria-chemoprevention settings.

Additionally, SAGE and the Malaria Policy Advisory Group (MPAG) requested an additional independent analysis of impact and cost-effectiveness be conducted to completement the analysis from Imperial College London. During the session, a team from Swiss Tropical and Public Health Institute (Swiss TPH) / Telethon Kids Institute presented plans for an analysis using a previously validated individual-based malaria model (OpenMalaria) that will be calibrated to vaccine efficacy estimates from Phase III trial data. Their modeling analysis will consist of the following stages: calibrating base models to trial site in the absence of vaccine introduction, estimating vaccination parameters for initial efficacy against infection, and then a validation exercise by matching predictions to trial data on vaccine efficacy against clinical malaria in sites not used for fitting. IVIR-AC welcomes an additional independent model and agrees that the proposed analysis plan is sound and appropriate and will help explore the uncertainty in R21/Matrix-M impact estimates and the potential drivers. Overall, IVIR-AC very much supports the need for 3-monthly vaccine efficacy estimates from the Phase III trial, as the currently available aggregate data at 12 and 18 months will not provide fine enough resolution to sufficiently estimate the waning of protection. The analysis will align with Imperial College London predictions for a range of vaccine deployment scenarios, make projections of impact in generic prevalence settings, and use similar assumptions on costs.

In general, across both modeling analyses, IVIR-AC suggests inclusion of more comprehensive sensitivity analyses for the economic analysis, including delivery costs, treatment coverage, time horizon, timing of doses/delay in receiving doses, and a budget impact analysis. Additionally, IVIR-AC notes that both modeling efforts are useful to guide implementation but do not replace detailed country-level analyses.

#### 1.3.2 Session 2: Meta-analysis of economic evaluations of vaccines

Evidence generated from economic evaluations is likely to play an increasingly important role in national-level vaccination-related decisions. Yet, many countries, especially but not exclusively low- and middle-income countries (LMICs), may not have data, resources, or expertise to conduct these evaluations. Until this capacity and expertise is realized, decision-makers from these locations may need to rely on evaluations from other settings. However, there are concerns about transferring evidence from one setting to another. Additionally, economic evaluations can provide overwhelming or conflicting information that can be challenging for decision-makers to interpret. Meta-analyses of economic evaluations (MAEE) of vaccines may allow pooling of results that may provide more reliable quantitative estimates and have been applied by multiple research teams [7], [8], [9], [10], [11]. In March 2021, IVIR-AC provided feedback and recommendations [12] on the value and limitations of performing MAEEs of vaccines using the incremental net benefit approach. IVIR-AC agreed that, in the absence of setting-specific economic evaluations, MAEEs could facilitate decision making, but noted the need for specific techniques for meta-analyses as applied to economic evaluations. To support this, an IVIR-AC subgroup on MAEE of vaccines was formed with the objective of facilitating an ecosystem utilizing MAEE of vaccines to support policy and decision-making globally, regionally, or in settings with limited data availability. At the meeting, the IVIR-AC subgroup presented on the gaps and challenges of MAEE with a work plan of proposed activities to facilitate such an ecosystem. Overall, IVIR-AC continues to support and agrees with the previous IVIR-AC recommendation that “high-quality, locally-produced and context-/country-specific economic evaluations should be maintained as the priority for decision-making over MAEE” [12].

During the session, the subgroup presented an overview of the various existing MAEE studies, gaps, and challenges. Current gaps and challenges include the following:

• There is a need to document heterogeneity in published MAEEs to explain sources of variation; additionally, there needs to be a standardized approach on how to address this heterogeneity (e.g., meta-regression).
• It is unclear how to appropriately weight studies within MAEEs, as uncertainty intervals within individual economic evaluations depend on how thoroughly the modelers have investigated uncertainty, rather than on inherent variability in the measure of cost-effectiveness.
• There is not currently a consensus on which indicator within economic evaluations should be meta-analyzed (e.g., cost, incremental net benefit, incremental cost-effectiveness ratio (ICER)).
• There is no existing formal evaluation of perception on the value of MAEE from the perspective of decision-makers.

The chair of the subgroup presented on the proposed research agenda. To understand the demand for and value of using MAEE to support policy and decision-making, globally, regionally, or in settings with limited resources, the subgroup plans to conduct a literature review to summarize potential use cases of MAEE, and qualitative interviews to identify suitable methodologies and contexts for application. Following these interviews, IVIR-AC recommends developing an informational product (e.g., a white paper) to outline the different contexts in which the decision-makers might find MAEE useful, including a general diagram (e.g., decision tree or checklist) on the key questions and decision points that should be addressed during the decision-making process.

To advance the methodology of MAEE, the subgroup will generate guidance on how MAEE analyses should be performed to minimize heterogeneity; IVIR-AC recommends that the proposed research to explore the influence of uncertainty on heterogeneity across studies in MAEE comprehensively reports the breadth of findings. The subgroup will also develop guidance on how weighting should be approached. IVIR-AC agrees that an exploration of different weighting approaches, particularly those based on study quality (including consideration of possible biases), is needed. Additionally, the subgroup will aim to explore other methodologic issues concerning MAEE. Then, the subgroup will develop an overall toolbox for using the MAEE results in country-specific decision-making. IVIR-AC recommends that the proposed toolbox includes products of the working group, existing guidance on conducting economic evaluations, methods, and applications of MAEE, including worked examples, and evidence on the rationale to use incremental net benefit as the metric of choice in MAEE.

Overall, IVIR-AC recommends a cautious discussion/reporting of the conclusions and generalizability of MAEE, given that the group of countries studies from which are likely to be included in MAEE (often high-income) and those looking to make decisions based on the MAEE results are likely to be quite different in terms of disease burden, health system, socio-economic characteristics, and other drivers of cost-effectiveness. Additionally, IVIR-AC strongly urges the inclusion of meta-analyses of both incremental costs and incremental effects alongside incremental net benefit as part of the standard protocol for MAEE and suggests that a mid-point check-in between the subgroup and IVIR-AC focal points be undertaken in advance of the September 2024 IVIR-AC meeting to assess progress on the proposed research agenda and the potential need for further refinement.

#### 1.3.3 Session 3: Global analysis estimating the impact of vaccination over 50 years

A project team within WHO IVB has been conducting an analysis that quantifies the historical impact (i.e., deaths and DALYs averted) of childhood vaccination from 1974 to 2024 for 11 diseases using the analytical framework they have developed for the Immunization Agenda 2030 (IA2030) vaccine impact estimates [13]. This analysis will be used to support advocacy efforts in celebration of the 50th anniversary of the Expanded Program on Immunization (EPI@50) during World Immunization Week (24–30 April) and World Health Assembly in 2024. IVIR-AC was asked to provide feedback on methodologic updates to the analytical framework of IA2030, including the non-linear impact function and imputation method for countries not modelled by the Vaccine Impact Modeling Consortium, as well as the strategies planned for communication of results.

The project team has received ongoing feedback on their analytic framework during previous IVIR-AC meetings. Since receiving feedback on modeling analyses from IVIR-AC in September 2023, the project team has extended the analytic framework methodology. During the session, the team presented on the inclusion of non-linear models for vaccine impact. This framework adaptation was incorporated as, in some cases, vaccination impact diminishes at higher levels of coverage. For each country and vaccine pair, three alternative functions were fit to cumulative deaths averted per fully vaccinated person using maximum likelihood estimation and the most appropriate function was selected via corrected Akaike information criterion. The team also presented on their evaluation of predictors of heterogeneity in vaccination impact between countries using time series models for each country and vaccine activity including, but not limited to, the following predictors: vaccination coverage, Gini coefficient, health spending, malnutrition, maternal mortality, and access to clean water and sanitation. The team then used model fits to impute vaccination impact in countries without estimates. Overall, IVIR-AC found the application of the non-linear impact estimation and geographical imputation approaches sound and appropriate for the EPI@50 analysis presented.

The project team also presented on plans and considerations for communication of the EPI@50 analysis in collaboration with stakeholders from WHO, Gavi, the Bill & Melinda Gates Foundation, and UNICEF.

The project team noted that the number of deaths and cases averted by vaccines over 50 years is large and will require careful framing to ensure comprehensibility. IVIR-AC recommends that results should capture the largely qualitative nature of the estimates as well as historical differences in vaccination programs across WHO regions, including baseline rates of respective diseases, timing of introduction, types of vaccines, and target age of vaccination, among others, that may have contributed to different absolute and relative impacts over the last 50 years. IVIR-AC also suggests that framing the analysis as “over the last 50 years” rather than “a 50-year analysis” may help to acknowledge that regional differences exist between the historical introduction and availability of vaccination programs.

Additionally, the project team notes that it will be critical to message who benefits from vaccination (e.g., rather than reporting the metric of ‘years of life lost’, the team proposed presenting ‘improved survival rates of children to their 5th birthday’) and that this analysis is retrospective and celebratory; however, it should simultaneously reinforce the need for sustained efforts and encourage vaccination. IVIR-AC agrees that, while celebrating the large historical health gains of childhood immunization, careful nuance is required to simultaneously communicate the need to continue high levels of vaccination, with emphasis placed on strategies to continue to reach unvaccinated children and on the message that any future reductions in coverage will lead to a loss of health gains.

The presented updated analysis from the project team allows the elucidation of factors associated with high impact of vaccination, which could potentially be used to advocate for resources to improve the effectiveness and efficiency of vaccination programs. In established programs, the impact of each additional dose lessens over time; and with increasing coverage, however, this should not lead to complacency towards uptake. Therefore, the project team proposed using the positive language of inclusion, such as finding and protecting those who have been missed by vaccination. Overall, with respect to plans for communication, IVIR-AC also acknowledges that care needs to be taken when communicating complex methods to a variety of audiences and that communication of results should capture stratification by WHO region, epidemiological characteristics of disease (e.g., age), and other equity domains (e.g., economic status or healthcare access).

#### 1.3.4 Session 4: Modeling the impact of different dose schedules for RTS,S malaria vaccine in seasonal settings

The RTS,S/AS01 vaccine is recommended to reduce *P. falciparum* malaria among children living in areas with endemic malaria transmission. Clinical trials have shown that the vaccine given in a four-dose schedule (three monthly primary doses and a later fourth dose) provides protection; however, the ideal schedule for optimal effectiveness, including the interval between third and fourth doses and the added benefit of seasonal or hybrid over age-based schedules, is unknown. The current WHO recommendation recognizes this knowledge gap and states that there can be flexibility in vaccination schedules to optimize fourth dose coverage. However, as countries are preparing for vaccine introduction, and planning vaccination schedules and delivery strategies, additional information that can be learned through mathematical modeling of different approaches can provide helpful insight to the decision makers.

In this session, two teams (Swiss TPH / Telethon Kids Institute and PATH) presented their modeling analyses on the public health impact of timing of the fourth dose across various settings and schedules. Overall, IVIR-AC agrees that both models used are generally well-suited to address the questions at hand and concludes that the models support the notion that there is currently insufficient evidence to strongly advocate for any specific timing of a fourth dose. The team from Swiss TPH / Telethon Kids Institute presented results from an analysis [14] using an individual-based malaria transmission model (OpenMalaria) to model potential timing of the fourth dose between 15 and 27 months among different transmission intensities and age patterns of severe disease in perennial transmission settings. The team also conducted sensitivity analyses on assumption of initial efficacy of fourth dose at 15 months of age, under different coverage assumptions. Overall, their modeling results suggest that the primary dose series is responsible for between 70 and 90 % of the total clinical cases averted and between 60 and 90 % of the severe cases averted; however, a fourth dose given 6 to 18 months after the third dose could avert additional malaria cases and deaths compared with the primary series alone. Additionally, fourth dose coverage is a crucial driver for added impact beyond the primary series. IVIR-AC notes that there are likely trade-offs between coverage (related to whether or not the dosing schedule is aligned with second dose of the measles-containing vaccine) and vaccine efficacy pertaining to differences in the time interval between third and fourth doses.

A team from PATH presented results from an analysis [15] of the impact of fourth dose of RTS,S/AS01 timing across settings of seasonal malaria transmission via an age-based individual-based malaria transmission model (i.e., the Imperial College London model described in Session 1). Their results suggest that, when modeled under the same coverage, dose number, and efficacy assumptions, various RTS,S schedules provided largely similar protection against clinical and severe malaria in settings with seasonal malaria transmission. However, the impact of various schedules differs in ages that see the highest reductions in clinical and severe cases; additionally, impact differs when considering potential improved efficacy of a 12-month dose spacing interval. The outputs of this exercise, however, should be considered in the context of understanding overall patterns rather than as directly guiding country-specific prioritization, and that modeling alongside local data should be used where available and may be particularly important to inform RTS,S introductions. Key considerations for countries will need to include delivery system ability to achieve high coverage, age-specific burden of disease in that area, other interventions in use, and the degree of seasonality. IVIR-AC acknowledges that the modelled results do present some additional evidence of the increased impact of seasonal delivery of the primary series (in settings with highly seasonal transmission), but this increased impact would have to be evaluated against potential lower coverage associated with seasonal delivery.

In general, across both models, IVIR-AC notes that there is need for more systematic exploration (and generation) of evidence and associated uncertainty of vaccine efficacy, particularly of waning and boosting, and how this differs with fourth dose timing. Additionally, models should more systematically explore in what realistic scenarios of waning and boosting of vaccine efficacy would vaccine impact be sensitive to the timing of the fourth dose and the program schedule. Modeled results should additionally include reporting on differences in incremental burden averted through delivery of the fourth dose when assessing differences in impact due to timing of the fourth dose. Finally, to generate sufficient evidence to explore differences in vaccine efficacy pertaining to fourth dose timing as well as the minimal interval (e.g., 3 months) between third and fourth doses, IVIR-AC recommends conducting prospective studies (e.g., trials) in addition to post-implementation monitoring.

### 1.4 Recurring themes

Across sessions, recommendations and discussion points consistently emphasized that (1) access to available data necessary for analyses must be prioritized for timely access to all available data sources; and (2) full explorations of uncertainty should be approached systematically, including explorations of statistical uncertainty of results, choice of parameters, modeling assumptions, and uncertainty contained within available data sources.

IVIR-AC will next convene from 10 to 13 September 2024.

## 2 WHO author disclaimer

Philipp Lambach and Mitsuki Koh work for the World Health Organization (WHO). The authors alone are responsible for the views expressed in this publication and they do not necessarily represent the decisions, policy or views of the WHO.

## CRediT authorship contribution statement

**Philipp Lambach:** Conceptualization, Project administration, Supervision, Writing – review & editing. **Sheetal Silal:** Investigation, Supervision, Writing – review & editing. **Alyssa N. Sbarra:** Conceptualization, Writing – original draft. **Mitsuki Koh:** Project administration, Writing – review & editing. **Rakesh Aggarwal:** Investigation, Writing – review & editing. **Habib Hasan Farooqui:** Investigation, Writing – review & editing. **Stefan Flasche:** Investigation, Writing – review & editing. **Alexandra B. Hogan:** Investigation, Writing – review & editing. **Sun-Young Kim:** Investigation, Writing – review & editing. **Kathy Leung:** Investigation, Writing – review & editing. **William J. Moss:** Investigation, Writing – review & editing. **Patrick K. Munywoki:** Investigation, Writing – review & editing. **Allison Portnoy:** Investigation, Writing – review & editing. **Meru Sheel:** Investigation, Writing – review & editing. **Xuan-Yi Wang:** Investigation, Writing – review & editing.

## Declaration of competing interest

The authors declare the following financial interests/personal relationships which may be considered as potential competing interests: S. S. was supported by the World Health Organization for this work. A. N. S. was financially supported by the World Health Organization for this work, and is additionally supported by the Bill & Melinda Gates Foundation, Gavi, the Vaccine Alliance, and the National Institutes of Health. A. B. H. was supported by the Australian National Health and Medical Research Council for this work, is additionally supported by PATH, the World Health Organization, and Gavi, the Vaccine Alliance, and has received consulting fees from the Australian NSW Ministry of Health, WHO Europe and Asian Development Bank. W. J. M. is supported by the National institutes of Health. A. P. is supported by Imperial College London, the Bill & Melinda Gates Foundation, the World Health Organization, and the University of Oslo. X. W. has served as a Data Safety Monitoring Board member for several China-made COVID-19 vaccines. S. S., A. N. S., and A. B. H. report travel related support from the World Health Organization to attend previous IVIR-AC meetings. All other authors have no declarations.

## Data Availability

No data was used for the research described in the article.
