# Corrigendum to “Report from the World Health Organization’s immunization and vaccines-related implementation research advisory committee (IVIR-AC) meeting, virtual gathering, 26 February–1 March 2024” [Vaccine 42(15) (2024) 3379–3383]

**DOI:** 10.1016/j.vaccine.2024.07.003

**Published:** 2024-10-03

**Authors:** Philipp Lambach, Sheetal Silal, Alyssa N. Sbarra, Mitsuki Koh, Rakesh Aggarwal, Habib Hasan Farooqui, Stefan Flasche, Alexandra B. Hogan, Sun-Young Kim, Kathy Leung, William J. Moss, Patrick K. Munywoki, Allison Portnoy, Meru Sheel, Xuan-Yi Wang

**Affiliations:** a Immunizations, Vaccines and Biologicals, World Health Organization, Geneva, Switzerland; b Modelling and Simulation Hub, Africa, University of Cape Town, Cape Town, South Africa; c Centre for Global Health, Nuffield Department of Medicine, Oxford University, Oxford, United Kingdom; d Johns Hopkins Bloomberg School of Public Health, Baltimore, MD, United States; e Jawaharlal Institute of Postgraduate Medical Education & Research, Puducherry, India; f College of Medicine, QU Health, Qatar University, Qatar; g Centre for Global Health, Charite, Berlin, Germany; h School of Population Health, University of New South Wales, Sydney, Australia; i Seoul National University, Seoul, South Korea; j School of Public Health, The University of Hong Kong, Hong Kong SAR, China; k Kenya Medical Research Institute, Centre for Global Health Research, Nairobi, Kenya; l Department of Global Health, Boston University School of Public Health, Boston, United States; m Center for Health Decision Science, Harvard T.H. Chan School of Public Health, Boston, United States; n School of Public Health, Faculty of Medicine and Health, University of Sydney, Sydney, Australia; o Shanghai Institute of Infectious Disease and Biosecurity, Fudan University, Shanghai, China

The authors regret an incorrect affiliation (j) for co-author Dr. Kathy Leung, which should be corrected to School of Public Health, The University of Hong Kong, Hong Kong SAR, China.

The authors would like to apologise for any inconvenience caused.

